# Intraductal Tissue Sampling Device Designed for the Biliary Tract

**DOI:** 10.1109/JTEHM.2021.3057234

**Published:** 2021-02-04

**Authors:** Malay S. Patel, Matthew D. Carson, Eric J. Seibel, Lucas R. Meza

**Affiliations:** 1 Department of Mechanical EngineeringUniversity of Washington 7284 SeattleWA98195USA; 2 Human Photonics LaboratoryUniversity of Washington 7284 SeattleWA98195USA

**Keywords:** Biliary biopsy, endoscopic biopsy device, finite element analysis, helical buckling, NiTi, superelastic alloy

## Abstract

Clinical sampling of tissue that is read by a pathologist is currently the gold standard for making a disease diagnosis, but the few minimally invasive techniques available for small duct biopsies have low sensitivity, increasing the likelihood of false negative diagnoses. We propose a novel biopsy device designed to accurately sample tissue in a biliary stricture under fluoroscopy or endoscopic guidance. The device consists of thin blades organized around the circumference of a cylinder that are deployed into a cutting annulus capable of comprehensively sampling tissue from a stricture. A parametric study of the device performance was done using finite element analysis; this includes the blade deployment under combined axial compression and torsion followed by an axial ‘cutting’ step. The clinical feasibility of the device is determined by considering maximum deployment forces, the radial expansion achieved and the cutting stiffness. We find practical parameters for the device operation to be an overall length of 10 mm and a diameter of 3.5 mm for a $50~\mu \text{m}$ blade thickness, which allow the device to be safely deployed with a force of 10N and achieve an expansion over 3x its original diameter. A model device was fabricated with these parameters and a $75~\mu \text{m}$ thickness out of a NiTi superalloy and tested to validate the performance. The device showed strong agreement with an equivalent numerical model, reaching a peak force within 2% of that predicted numerically and fully recovering after compression to 20% of its length. ***Clinical and Translational Impact Statement***–This pre-clinical research conceptually demonstrates a novel expandable device to biopsy tissue in narrow strictures during an ERCP procedure. It can greatly improve diagnostic tissue yield compared to existing methods.

## I. Introduction

Endoscopic retrograde cholangiopancreatography (ERCP) is a medical procedure that is applied to solve problems associated with the pancreatobiliary system. Part of this procedure involves the procurement of a biopsy from either the pancreatic or biliary ducts. A bile duct biopsy procedure is one in which a sample of tissue or cells is safely extracted to diagnose disease, which is often a preliminary step before determining if the patient needs to have a surgical resection. The tissue sampling technique must have high sensitivity for detecting malignancy while maintaining sufficient specificity. As with any procedure, ERCP-based sampling techniques should be safe, simple, and relatively inexpensive so they can be widely used. The remote, narrow-branched structure of the bile duct (ID < 6 mm) makes it difficult to view constrictions or maneuver the tool to perform the sampling procedure. The current standard for biopsy guided acquisition is use of fluoroscopy to direct the device to areas in question. Studies have demonstrated that biliary stricture sampling methods acquire miniscule amounts of tissue and frequently contain insufficient cellularity [1], often leading to false-negative diagnoses [2]. The most common biopsy method in the bile duct is brush sampling of cells for cytology [3]. In this procedure, a guidewire steers a brush passed into a duct which is often constricted. When the guidewire is pushed and pulled, the epithelial cells on the duct walls get trapped in the dense bristles and in this way the sample can be obtained. However, the sensitivity of standard biliary brushings is often low, around 30% – 60% [4]. It has been suggested that the low sensitivity of brush cytology is mainly due to inadequate cellular sampling, which may be because many malignancies compress the biliary tree from outside [5] or because of the fibrolamellar growth of many bile duct tumors [6]. A study conducted by de Bellis et. al. on the use of brush cytology in combination with stricture dilation found that stricture dilation does not improve the sensitivity of brush cytology [5]. In general, it is preferred that epithelial to deeper submucosal tissues be obtained for proper tissue diagnosis.

**Fig. 1.**
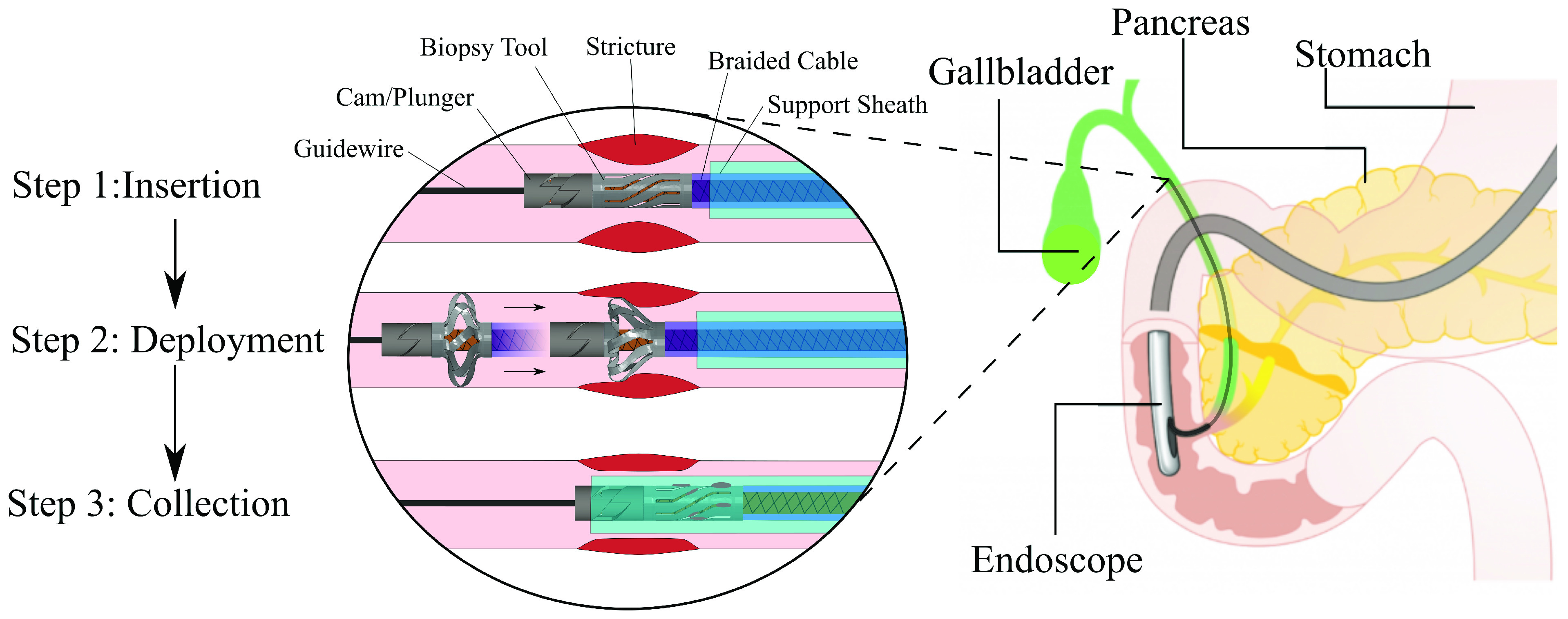
Working schematic of the intraductal biopsy tool during insertion, deployment, and collection of tissue from a constriction in the biliary duct.

A less common method of sampling tissue in the biliary ducts is the forceps biopsy procedure. The forceps are a pair of sharp-edged jaws controlled by a coaxial wire within a flexible shaft. These forceps can be opened or closed by pressing and releasing the knob at the end of a shaft which is present in the hands of the operator. In operation, the two sharp edged cups of the forceps are opened, pressed against tissue in question, closed, and then the device is quickly pulled back to rip out a small piece of tissue from the bile duct wall. This technique is more time consuming and more technically challenging than brushing and thus is less commonly used [2]. The forceps are ill-designed for small ducts because the opening process expands the tool which restricts access to the constrictions where diseases often reside. Further, the rigid and linear nature of the forceps preclude directed tissue sampling from straight luminal surfaces. For example, biopsying tissue behind an angled bile duct cannot be performed as the device cannot be angled acutely. In some patients, biopsies are not feasible as the positioning of the endoscope below the stomach does not allow for passage of the standard-sized forceps out of the accessory channel due to the thick and thus more rigid nature of the device [7] (see Fig. 1). A study reported by Korc and Sherman stated that biopsy forceps only tend to sample the rim of the tumor which can affect the adequacy of the tissue collection [2]. An earlier study performed by Savader, Prescott, Lund and Osterman on intraductal biliary biopsy concluded that brush cytology and fluoroscopy guided forceps had zero sensitivity for diagnosing bile duct cancer (cholangiocarcinoma), while sensitivity increased to 27% when using direct optical imaging of forceps within the bile duct [8]. More recently a custom single-operator cholangioscope that provides this direct optical imaging and a smaller-sized forceps system were developed, and sensitivity has been improved to be greater than brush biopsy [2], [9], [10]. The advent of second generation cholangioscopes and laser-based imaging catheters may provide greater improvements in sensitivity and specificity but have not provided satisfactory diagnostic yield of tissue during cholangiopancretoscopy, which may be addressed with new innovative biopsy tools [11], [12]. Since major life-changing decisions are made on the results of these procedures, there is room for improvement in reducing complexity and cost while raising sensitivity and specificity of these biopsy procedures. Our current techniques are limited in their ability to provide reassurance in the management of benign disease or targeted therapy in the face of malignancy, leading to both incorrect disease diagnoses and increased healthcare utilization due to repeat procedures.

We propose a novel biopsy tool design that overcomes the drawbacks associated with the current methods for the bile duct biopsy procedure. The biopsy tool consists of thin, circumferential blades supported by a hollow sheath and actuated by a helical plunger (Fig. 1). Once a constriction or suspicious region is located, the tool is deployed to form a cutting annulus capable of uniformly collecting tissue samples around the circumference of the duct. It is actuated by pulling a metal braided cable sleeve to contact the plunger and compress and twist the tool along its axis. This causes the blades to buckle laterally, which deploys them outward and presses them into the ductal tissue. The deployed tool is pulled by both the cable sleeve and support sheath to cut the constricted ductal tissue section. After cutting, the cable sleeve is released to allow the elastic blades to close and trap tissue within them. Once the procedure is completed, the biopsy tool is retracted into an optional sheath for removal and then tissue analysis by a pathologist. This process is illustrated in Fig. 1 for the important case of an indeterminate stricture. To validate this design, we have performed a parametric study of the tool using the commercial finite element analysis (FEA) package Abaqus. In this engineering analysis, we have mapped the performance of the tool across various metrics including size, deployment force, expandability, and maximum cutting force. After determining a suitable design, we fabricated the device at scale out of a nickel titanium (NiTi) superalloy and experimentally validated the force response, expansion and recoverability under combined axial compression and torsion loading.

## II. New Biopsy Device Design

### A. Design Requirements

There are three requirements for the tool to be effective in ERCP operation: 1) a low (<30N) actuation force, 2) a 2-3x expansion ratio and 3) minimal blade deflection during cutting. For the first requirement, the maximum force required to actuate the tool should be less than the force which will cause the plastic support catheter to malfunction which is >30 N depending on components selected from the clinic. Second, the tool will need to expand in the bile duct to provide a radial force and introduce tension to the biliary luminal surface; this will ensure the device cuts instead of simply deforming the tissue. The tool will initially have a diameter of 3.5 mm so that it can fit through a constriction that is temporarily enlarged with a balloon catheter if needed. The expansion of the device will introduce any necessary additional expansion of the duct. An expansion of 2-3x is estimated to be required to safely cut tissue based on clinical experience. Finally, to ensure that the tool can cut efficiently, the blades of the tool will need to withstand a certain force without significant deflection. Because the exact cutting force is presently unknown and will highly depend on the sharpness of the blades, this study will solely look at the “cutting stiffness” of the tool, or its ability to withstand cutting forces while sustaining minor deflections. It is important to note that we are not simulating the cutting of tissue, only applying a loading state similar to that experienced during cutting, although without the interaction between the tool and the surrounding tissue.

### B. Device Design

The tool design consists of thin angled blades organized along the circumference of a hollow cylinder. The device is deployed to form a cutting annulus capable of uniformly collecting tissue samples around the circumference of a duct. The proximal end of the tool is rigidly fixed to a plastic support catheter which is inserted into a duct over a custom metal braided cable sleeve. Once a constriction is located, a helical plunger attached to a cable sleeve is used to compress and twist the distal end of the tool. This combined twisting and compression along the distal end of the tool causes the blades to deploy via buckling. The deployed tool with compressing sleeve is pulled to cut the inner lining of the biliary duct across the circumference of the stricture. Upon releasing the cable sleeve, compressive pressure is released, and the blades relax and close. The sliced tissue and cells are collected by sliding another sheath around the device to fully contain the biopsied tissue and then removing the device. This process is illustrated in Fig. 1.

**Fig. 2.**
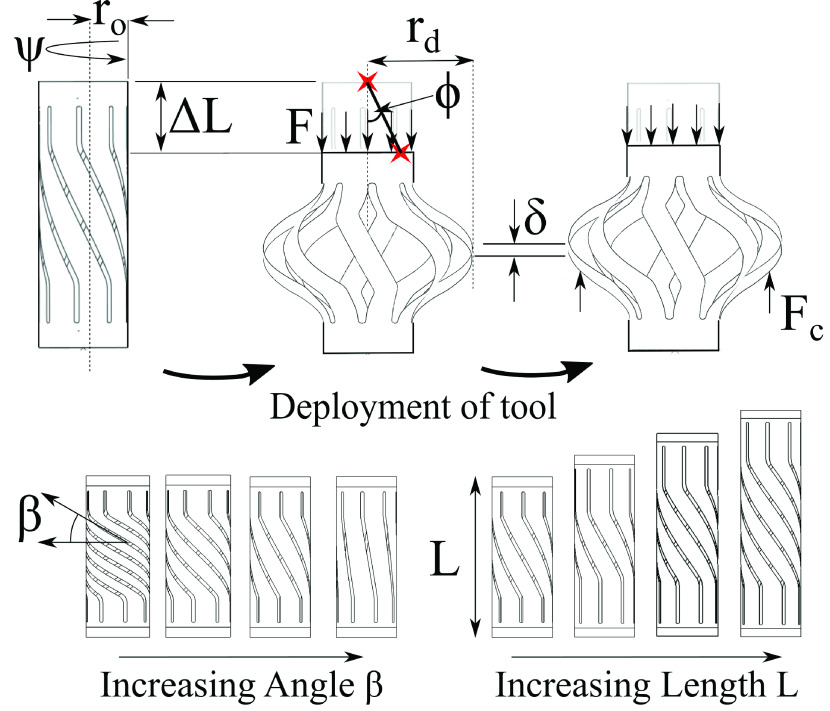
This illustrates the novel design of the tool and shows the relevant parameters considered for modeling. Different tool designs are obtained by varying the $\phi, \beta $, and $L$ parameters.

The relevant device dimensions are the length of the tool ($L$), the angle of slots and blades ($\beta$), and the initial tool radius ($r_{o}$). The tool is simultaneously twisted by an angle $\psi $ and axially displaced by a distance $\Delta L$, which induces a reaction force ($F$) from the tool, also known as the deployment force. This causes the blades to extend to a new radius ($r_{d}$), which is measured using the maximum displacement of the blades. The axial displacement and angle of twist can be reduced to a single parameter, namely the helical displacement angle ($\phi$), to better quantify the numerical results. The helical displacement angle is related to the twist and axial displacement by the equation $\phi =\tan ^{-1}\left ({\frac {r_{o}\psi }{\Delta L} }\right)$. The deployed blades are then subjected to a cutting force ($F_{c}$) which causes a corresponding maximum blade deflection $(\delta)$. The cutting force is only applied parallel to the axis of the tool since it works by being pulled along the bile duct as explained above. These parameters and the corresponding device operation are illustrated in Fig. 2.

This tool design can be optimized by changing the length, the initial angle of the blades and the helical deployment angle. This design was chosen due to its simplicity and ease of manufacturing, both of which are achieved because the tool is made from a single tube. It is also highly compatible with other components of the system such as the cable sleeve and sheath arrangement and requires only a combination of torsion and axial displacement to deploy it, making it versatile and easy to operate.

### C. Parametric Study

A parametric study was performed for the device to find an optimal combination of parameters for efficient operation. The three parameters that were varied are: 1) the length of tool ($L$), 2) the angle of the blades ($\beta$) and 3) the helical displacement angle ($\phi$). For the design study, the above parameters were varied individually while keeping the other parameters constant, including a wall thickness of $50~\mu \text{m}$. The chosen parameters are specified in Table 1.

**TABLE 1:** Geometry Variation for Parametric Study

Quantity	Conversion from Gaussian and CGS EMU to SI ^a^
Tool Length	L_1_ = 8 mm, L_2_ = 10 mm, L_3_ = 12.5 mm, L_4_ = 15 mm
Blade Angle	$\beta _{1}= 3 0^{\circ },\beta _{2} = 45^{\circ },\beta _{3} = 60^{\circ },\beta _{4} = 75^{\circ }$, $\beta _{5} = 80^{\circ }$
Helical Displacement Angle	$\phi _{1}= 24^{\circ }$, $\phi _{2} =34^{\circ }$, $\phi _{3} = 42^{\circ }$, $\phi _{4}=48^{\circ }$, $\phi _{5} = 53^{\circ }$

The goal of this study is to quantify the performance of the tool during deployment and cutting. This includes measuring the reaction force ($F$) necessary to deploy the tool using the cable sleeve, measuring the expansion ratio ($r_{d}/r_{o}$) during deployment, and measuring the deflection of the blades ($\delta /L$) during cutting. These relate to the required design parameters of the low actuation force, the large expansion ratio and the minimal cutting deflection described in the previous section. For all tests, the devices were taken to a maximum displacement of $\Delta L=2 mm$ during the deployment step, and a maximum cumulative cutting force of $F_{c}=1N$ was applied to the blades during the cutting step.

## III. Numerical Analysis

### A. Model Setup

Numerical modeling was performed to quantify the behavior of the tool subject to loading conditions similar to those faced during a biopsy procedure. Modeling was done using the commercial Finite Element Analysis (FEA) software Abaqus with an explicit dynamics solver to capture the non-linear buckling behavior of the device. The high shell thickness-to-tube radius ratio (~1/25) necessitated the use of shell elements to accurately predict the stress state and deflection of the blades [13].

**Fig. 3.**
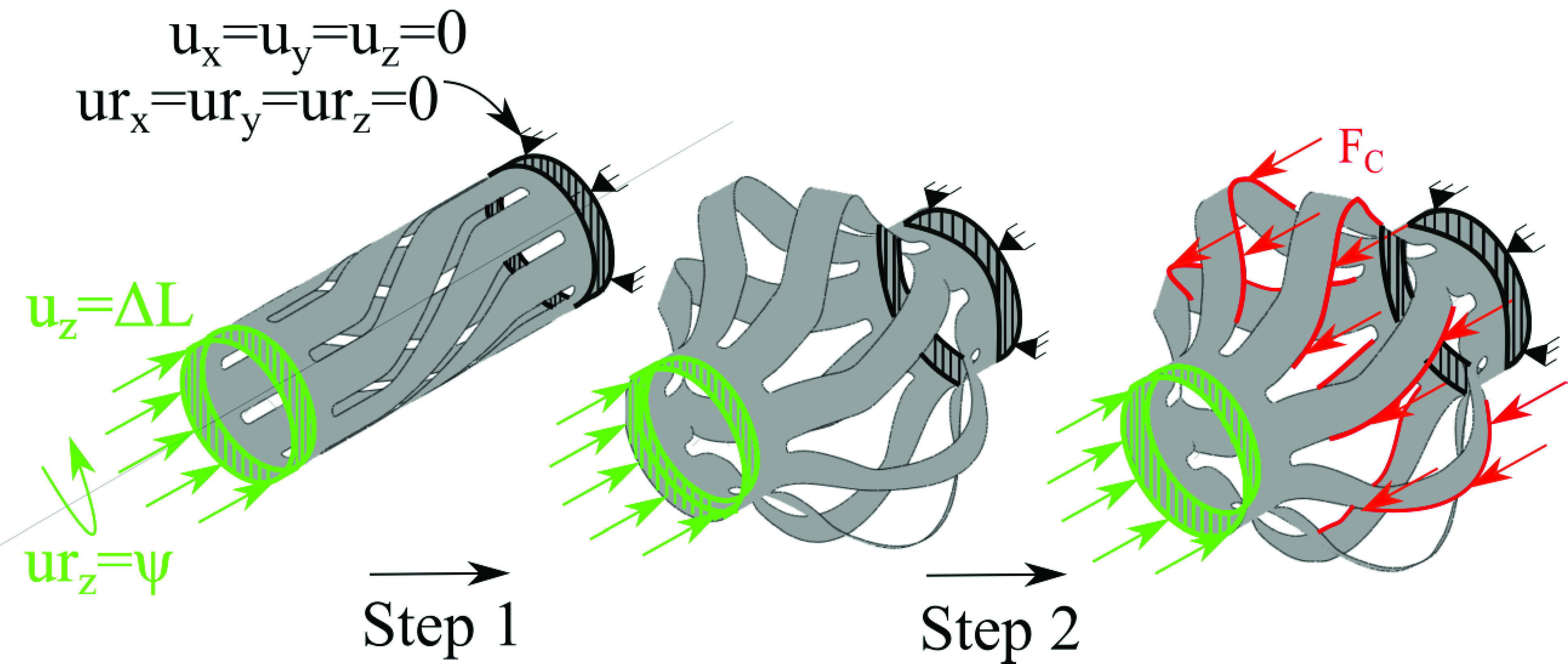
Numerical setup of the tool showing the boundary conditions and loads applied.

Three steps were included in the numerical model to capture the deployment, cutting and retraction of the device. In all the steps, the proximal edge is held fully fixed and has no degrees of freedom, as shown in black in Fig. 3. In the first step, the distal edge is displaced and rotated about the z-axis, as shown in green in Fig. 3. In the second step, the displacement of the distal edge is held constant and a cutting force is applied on the blade edge in the z-direction, as denoted by the red arrows in Fig. 3. In the third step, the force, axial displacement, and rotation are gradually reduced, and the tool is allowed to retract to its original state. Mass scaling was incorporated in the numerical model to speed up the simulation, and measures were taken to ensure the kinetic energy of the system was minimal compared to the total energy.

### B. Constituent Material Properties

The tool is modeled out of a superelastic shape memory alloy NiTi, which has been chosen due to its biocompatibility, extreme flexibility, and its ability to undergo large deformations without losing the ability to recover upon unloading [14]. NiTi has a very high transformation strain of 6% as compared to other metals for which the plastic strain limit is of the order of 0.1% [14] and can therefore withstand a high strain while still being able to recover. It exhibits a hysteresis in its loading behavior due to a reversible change from an austenite phase to martensite phase during deformation [14], [15], [16]. In its undeformed state, the material is in an austenite phase and upon straining it transforms into a martensite phase. This transformation is reversible, but the forward transformation occurs at a higher stress than the reverse transformation, leading to a hysteresis in the stress-strain behavior. The salient behaviors of the tool for this design study are only dependent on the forward transformation, particularly because the tool is intended to be a single use device, so only values for the forward transformation are used in modeling the material. These properties are shown in Table 2.

**TABLE 2:** Material Properties of Nitinol

Constant	Symbol	Value
Austenite Young’s Modulus	$E_{A}$	54 GPa
Poisson’s Ratio	$\nu $	0.33
Starting austenite-to-martensite stress	$\sigma _{s}^{AM}$	462 MPa
Finishing austenite-to-martensite stress	$\sigma _{f}^{AM}$	596 MPa
Ultimate Tensile Strength	$\sigma _{ult}$	895 MPa
Martensite Young’s Modulus	$E_{M}$	28 GPa
Finishing Austenite to Martensite	$\varepsilon _{f}^{AM}$	0.05
Poisson’s ratio	$\rho $	6.45 g/cc

### C. Experimental Methods

**Fig. 4.**
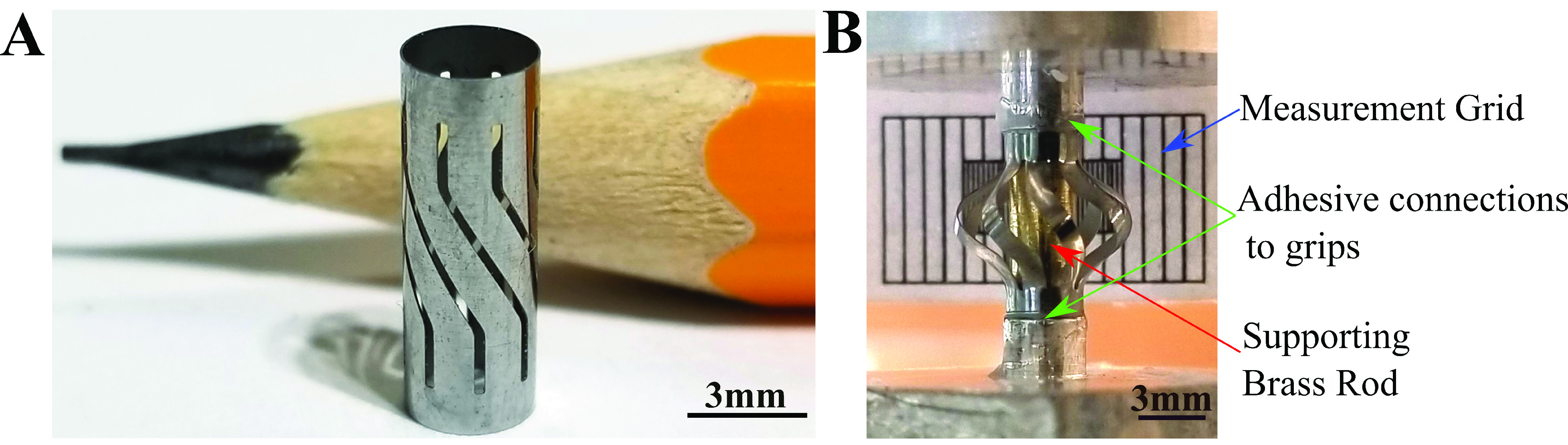
A) The to-scale fabricated NiTi biopsy device B) The device as set up in the compression-torsion mechanical testing system.

To validate the numerical model and to examine the experimental performance of the device, samples were fabricated at-scale out of a NiTi superalloy tube stock. The tube had a $3.5 mm$ ID, a $10 mm$ length and a $75~\mu \text{m}$ wall thickness. It should be noted that the thickness is different from the model device, and a numerical model with the experimental parameters was made for validation with the fabricated device. Samples were fabricated using a stepwise procedure that included Laser cutting, chemical cleaning and visual inspection by MeKo Laser Material Processing (Fig. 4A). The higher thickness tube was used due to constraints in availability and is not as optimal for an actual device due to the higher force required for deployment. For validation purposes, a corresponding numerical model matching these parameters was created.

A combined axial-torsion testing system (Instron 85215) was used to examine the mechanical performance of the device during the deployment of the blades (Fig. 4B). The two ends of the device were rigidly adhered to the grips of the torsion testing system and the fixture was stabilized by a brass rod going through its center. One end of the brass rod was connected to a threaded screw using an adapter which was connected to the upper grip on the machine. The other end of the rod was connected to a square block that was supported by the lower grip on the machine. Samples were compressed to a total displacement of $\Delta L=2 mm$ with a corresponding helical twist angle of $\phi =34^{\circ }$. The purpose of this step was to validate the deployability and recoverability of the device. No attempt was made to quantify or improve upon the sharpness of the blades.

## IV. Results

### A. Model Device Development

**Fig. 5.**
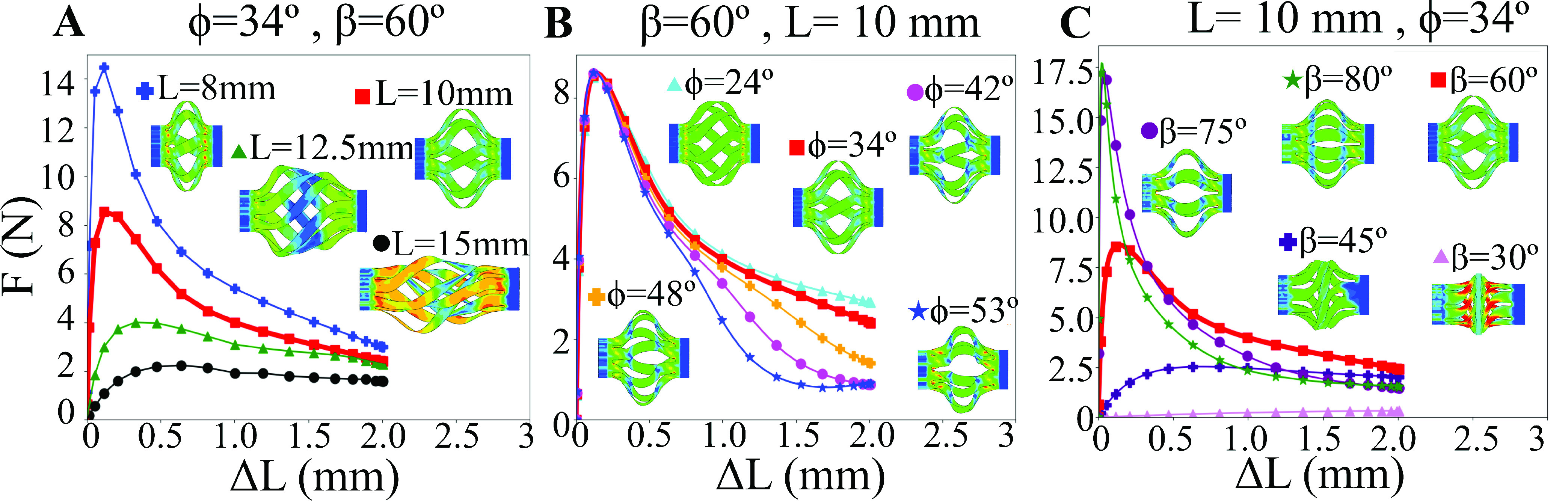
The reaction force of the tool to an applied displacement is shown for different parametric conditions. A) Increasing the length of the device decreases the peak load because it is a buckling reaction. B) Changing the helical displacement angle only affects the large-displacement behavior because it alters the buckled shape. C) Changing the blade angle has a pronounced effect on the peak load and final deformed shape because it changes the buckling mode. The result for $L=10 mm$, $\beta =60^{\circ }$ and $\phi =34^{\circ }$ is shown as a thicker red line in each plot.

The force required to actuate the tool ($F$), i.e. the deployment force, is shown as a function of the applied axial displacement ($\Delta L$) for different design parameters in Fig. 5. The three plots therein correspond to variations in the tool length ($L$), helical displacement angle ($\phi$) and blade angle ($\beta$). It is found that for all designs, the tool load response is primarily governed by the buckling response of the blades. All the device designs have a linear elastic behavior at the beginning of their displacement followed by either a drop or a plateau in the load corresponding to a transition to a buckled state. In all cases, the reaction force reaches its peak when the blades start to buckle. It should be noted that applying the force in this manner causes a torsional reaction force ($T$) that reaches a maximum of $3.5N/mm$ for the optimal device design. While this was not explicitly considered in this design, the chosen cable sheath to actuate the plunger must be stiff enough to resist this torsion without undergoing significant deflection.

Fig. 5a shows the effect of changing $L$ on the reaction force for a tool with $\beta =60^{\circ }$ and $\phi =34^{\circ }$. Here it is observed that the peak reaction force decreases as a function of increasing length. For $L=8 mm$ and $10 mm$ the force reaches a maximum of $14.5N$ and $8.6N$ respectively followed by a sharp drop in load after the initiation of buckling. As the tube length is increased, the sharp load drop after buckling is reduced or eliminated entirely, and the load plateaus once the maximum value is reached. The deployed biopsy tools are shown for different tool lengths in Fig. 5a. As the length is increased above $12.5 mm$, the buckling becomes highly nonuniform and the blades become entangled with each other. Only for lengths of $8 mm$ and $10 mm$ do the tools have a uniform buckling.

Fig. 5b shows the effect of changing $\phi $ on the reaction force for a tool with $\beta =60^{\circ }$ and $L=10 mm$. Changing the helical displacement angle has almost no effect on the peak reaction force, which reaches a value of approximately 8.6 N, but it does affect the reaction force of the tool at large displacements. The reaction force at a large $\Delta L$ is inversely proportional to the deployment angle. For $\phi =24^{\circ }$ the final reaction force is $\sim 3.5N$ while for $\phi =53^{\circ }$ it is $\sim 1.5N$. It can be observed in the deployed biopsy tools in Fig. 4b that the lower the displacement angle, the more “twisted” the end blade shapes are, meaning the blades do not buckle laterally outward but instead simultaneously buckle and twist to expose more of the cutting edge. While it is likely that this will affect the cutting ability of the tool, it is difficult to quantify the impact on cutting effectiveness absent any experimental validation.

Fig. 5c shows the effect of changing $\beta $ on the reaction force for a tool with $\phi =34^{\circ }$ and $L=10 mm$. It is observed that changing the blade angle has a drastic effect on the reaction force, with an increased blade angle corresponding to a sharply increased reaction force. For a blade angle of $\beta =30^{\circ }$, the peak force reached is less than 0.5 N, while a blade angle of $\beta =80^{\circ }$ reaches a peak force of over $17.5N$.

The deployed biopsy tools in Fig. 5c show the drastic change in tool shape for changing blade angle. For $\beta $ values of 30° and 45°, the blades buckle easily and remain orthogonal to the tool axis. As the $\beta $ angle is increased, the blades buckle progressively more laterally and remain increasingly parallel to the tool axis.

### B. Model Device Expansion Ratio

**Fig. 6.**
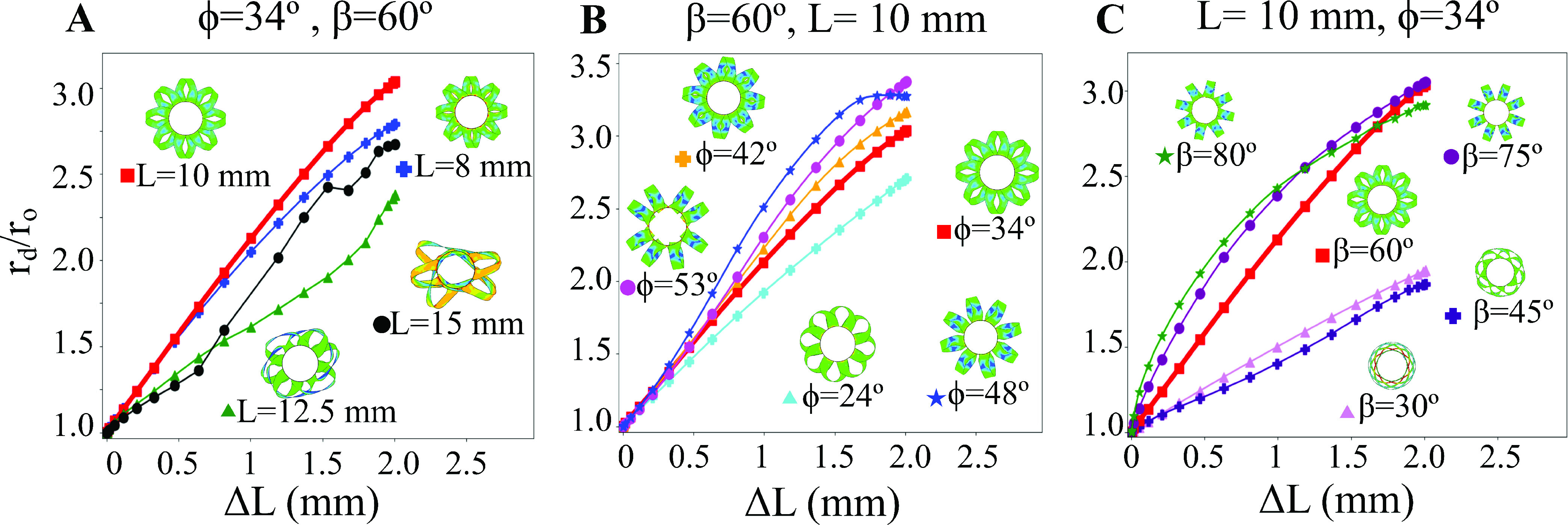
The tool expansion ratio achieved as a function of axial displacement is shown for different parametric conditions. A) Changing length only minorly affects the expansion, but this is complicated by the odd buckled states of the blades. B) Increasing helical displacement angle promotes the expansion of the device at the expense of the optimal blade angle. C) Changing blade angle can drastically affect the expansion when the blades take on a new buckled shape (e.g. for low blade angles). The result for $L=10 mm$, $\beta =60^{\circ }$ and $\phi =34^{\circ }$ is shown as a thicker red line in each plot.

To determine the ability of the tool to push against the bile duct during deployment, its expansion ratio ($r_{d}/r_{o}$) was measured as a function of the applied axial displacement ($\Delta L$). The expansion ratio, as shown in Fig. 6, was calculated by measuring the average maximum radial deflection of the blades during the deployment step. This can be seen in the axial view of the deployed tools, where the inner circle represents the initial tool shape and the blades can be seen stretching to a new deployed radius. The large expansion ratio of the tool is enabled by the large deflection of the blades during buckling.

Fig. 6a shows the effect of changing $L$ on the expansion ratio for a tool with $\phi =34^{\circ }$ and $\beta =60^{\circ }$. Changing the tool length from $L=8 mm$ to $10 mm$ leads to an increase in expansion ratio from $r_{d}/r_{o}\approx 2.75$ to $r_{d}/r_{o}\approx 3.05$ respectively at a displacement of $\Delta L=2 mm$. However, when the length increases to $12.5 mm$ or longer, the maximum expansion ratio of the tool drops to $r_{d}/r_{o}\approx 2.3-2.5$. As can be seen from the axial view of the deployed tools in Fig. 6a, only the $8 mm$ and $10 mm$ tools remain radially symmetric. As the length is increased, the buckling of the blades becomes non-uniform and there is no consistent deflection of the blades.

Fig. 6b shows the effect of changing $\phi $ on the expansion ratio for a tool with $L=10 mm$ and $\beta =60^{\circ }$. As the value of $\phi $ is increased, there is a corresponding increase in the maximum expansion ratio of the tool, which goes from $r_{d}/r_{o}\approx 2.75$ for $\phi =24^{\circ }$ to $r_{d}/r_{o}\approx 3.35$ for $\phi =53^{\circ }$ at a displacement of $\Delta L=2 mm$. This change in expansion ratio corresponds to the shift from a buckling and twisting behavior at $\phi =24^{\circ }$ to the purely lateral buckling for $\phi =53^{\circ }$.

This change in angle is readily seen in the axial view of the deployed tools in Fig. 6b.

Fig. 6c shows the effect of changing $\beta $ on the expansion ratio for a tool with $L=10 mm$ and $\phi =34^{\circ }$. As the blade angle is increased from $\beta =30^{\circ }$ to $\beta =60^{\circ }$, the expansion ratio increases from $r_{d}/r_{o}\approx 1.8$ to $r_{d}/r_{o}\approx 3.05$ respectively at a displacement of $\Delta L=2 mm$. Interestingly, the maximum expansion ratio does not increase beyond $r_{d}/r_{o}\approx 3.5$ for $\beta =75^{\circ }$ or $\beta =80^{\circ }$. From the axial view of the deployed tools in Fig. 6c, it can be readily observed that the buckled blades for $\beta \le 60^{\circ }$ are oriented orthogonally to the tool axis, while the $\beta \ge 75^{\circ }$ blades buckle laterally with no twist to change their orientation.

### C. Model Cutting Ability

**Fig. 7.**
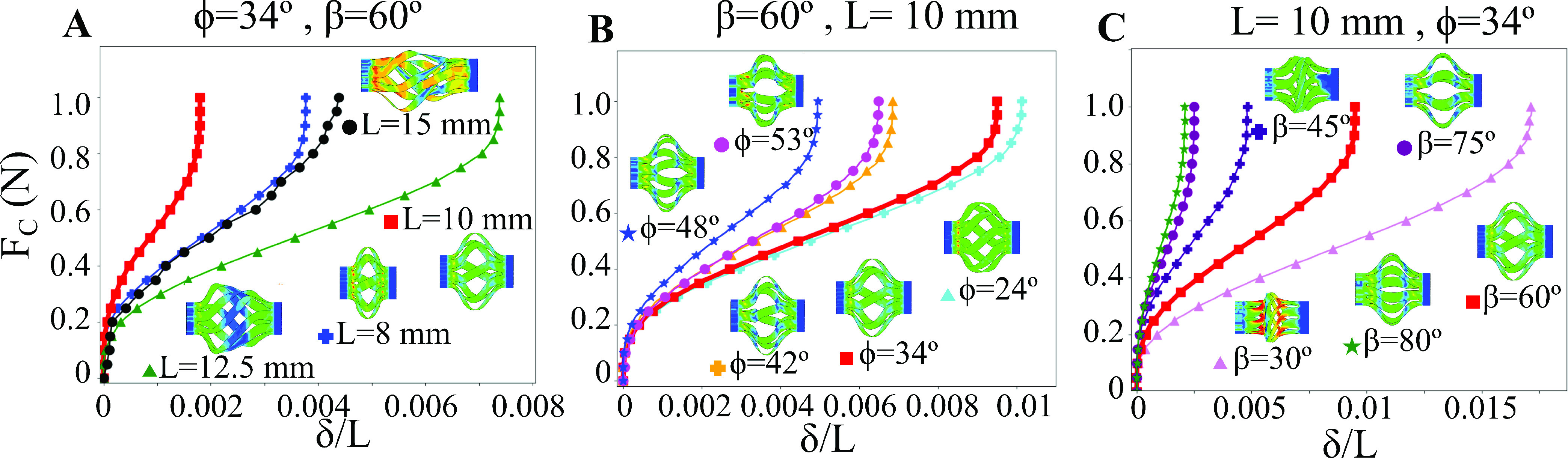
The deflection of the tool as a function of the applied cutting forces is shown for different parametric conditions. A) The $L=10 mm$ tool has the stiffest response because of the uniform buckling and lower blade deflection. B) Increasing the helical displacement angle will stiffen the device at the expense of a non-optimal blade angle. C) Increasing the initial blade angle will also stiffen the device at the expense of a non-optimal blade angle. The result for $L=10 mm$, $\beta =60^{\circ }$ and $\phi =34^{\circ }$ is shown as a thicker red line in each plot.

To quantify the effectiveness of the tool as a cutting device, the average blade displacement ($\delta$) was calculated as a function of a uniform applied axial force ($F_{C}$) along the edge of the blade. This response is shown in Fig. 7 for each of the parameters studied in the model up to a maximum total applied cutting force of $1 N$. The trends of the blade deflection can be divided into three stages. In the first stage, there is ‘stiff’ response of the blades, meaning they are highly resistant to deflection. There is little difference between the deflection of the different designs in this regime. In the second stage, which occurs after the force passes a threshold value of ~0.2N, there is a ‘flexible’ regime wherein the blades reorient, and their deflection abruptly increases. In the third stage, when the force passes a threshold of ~0.8N, the blades reorient and begin to stiffen again. This response is qualitatively similar for all tool parameters, but the stiffness and deflection vary considerably depending on the parameters.

Fig. 7a shows the effect of changing $L$ on the cutting reaction for a tool with $\beta =60^{\circ }$ and $\phi =34^{\circ }$. As the length is increased from $L=8 mm$ to $10 mm$, the relative deflection of the tool approximately doubles from $\delta /L=0.004$ to $\delta /L=0.002$ respectively for the maximum applied force of $1N$. As the length increases to $12.5 mm$ and greater, there is a monotonic decrease in the maximum deflection. Although this would suggest that longer tools are stiffer, the quantitative results are unclear due to the stochastic nature of the blade deflection for these designs. This is illustrated by the subsets in Fig. 7a, which show the symmetric deflection of the shorter tools and the sporadic deflection of the longer tools as they are pulled along the tool axis.

Fig. 7b shows the effect of changing $\phi $ on the cutting reaction for a tool with $\beta =60^{\circ }$ and $L=10 mm$. Here, the magnitude of the deflection decreases monotonically as $\phi $ is increased. It decreases from $\delta /L=0.01$ for $\phi =24^{\circ }$ to $\delta /L=0.0045$ for $\phi =48^{\circ }$ at the maximum applied force of $1N$. This means that applying a higher twist to the part can considerably stiffen the reaction, nearly 2$\times$ stiffer for the parameters examined here. This change in stiffness occurs because the blades for a larger applied $\phi $ are buckled laterally, meaning they are resisting deformation through bending of the blade. For smaller applied $\phi $, the blades are oriented orthogonally, which means they may be more effective at cutting but they resist the cutting force via blade twisting, making them more compliant. This is shown in the deformed blades in Fig. 7b.

Fig. 7c shows the effect of changing $\beta $ on the cutting reaction for a tool with $\phi =34^{\circ }$ and $L=10 mm$. There is no clear trend in the cutting stiffness with blade angle. The maximum deflection decreases from $\delta /L=0.0175$ for $\beta =30^{\circ }$ to $\delta /L=0.005$ for $\beta =45^{\circ }$ at the maximum applied force of $1N$. This increases to $\delta /L=0.01$ for $\beta =60^{\circ }$ before decreasing drastically again for both $\beta =75^{\circ }$ and $\beta =80^{\circ }$. This seemingly random trend has to do with both the change in deformation from bending to twisting of the deformed blades but also the fundamental change in buckling mode for the lower $\beta $ angles. There is only a small section of the blade that is buckled for $\beta =30^{\circ }$ and $\beta =45^{\circ }$, whereas the full blade has buckled in the other cases. This short buckled section means the blades will be inherently stiffer per unit length of the tool. The change in buckled shape can be observed in the deformed blades in Fig. 7c.

### D. Stress and Strain Distribution

**Fig. 8.**
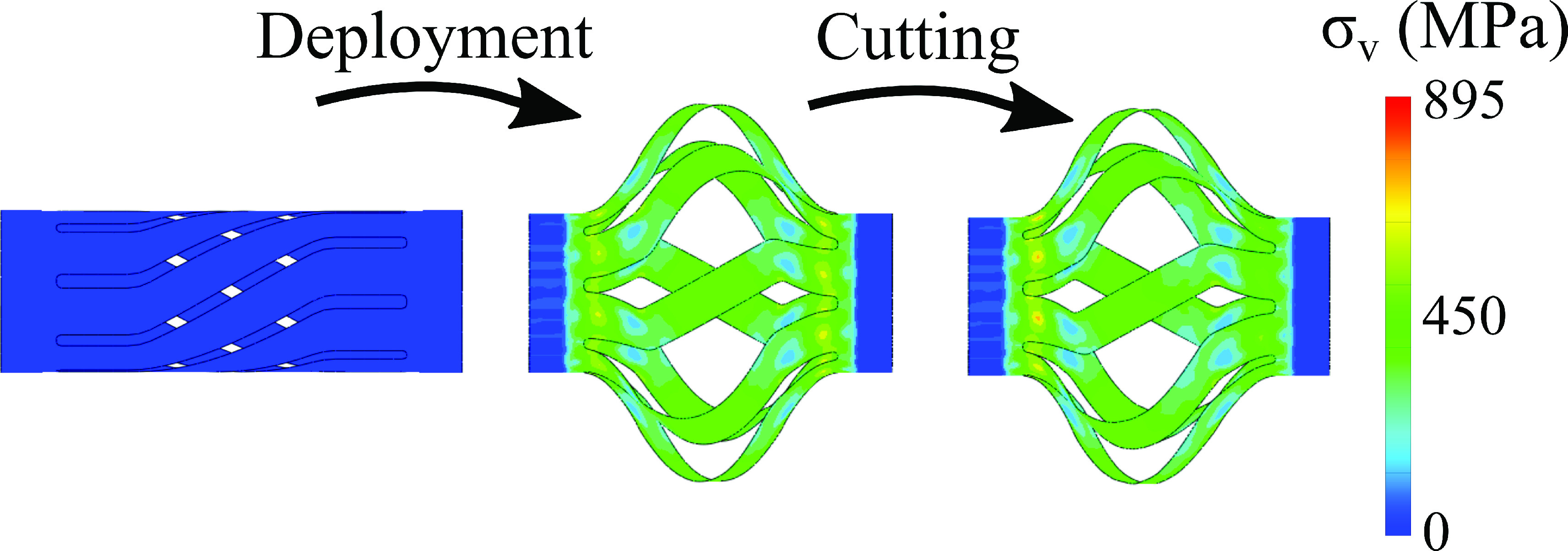
Stress in the device. The contour plot of the von-Mises stress during the deployment and cutting steps show that it does not exceed the ultimate stress of the NiTi.

A representative strain distribution is presented in Fig. 8 for the deployment and cutting step in the analysis. These are shown for a tool with length $L=10 mm$, blade angles of $\beta =60^{\circ }$ and helical displacement angle of $\phi =\mathrm { }34^{\circ }$. To quantify the behaviors in the deformed states of the tool, the von Mises stress ($\sigma _{v}$), or equivalent stress, is shown for the tool. To recount, the superelastic NiTi is recoverable if its peak stress does not exceed the ultimate stress of $\sigma _{ult}=895MPa$ [14], [15], [16].

The maximum stress in the model does not exceed 645 MPa during the deployment step, and it occurs at the upper and lower ends of the blades. This stress is on the order of the final austenite to martensite transition stress $\sigma _{f}^{AM}$. Similar high stresses are observed in the centers of the blades where they undergo the maximum bending, but these stresses do not exceed 486 MPa. In the cutting step, it is observed that the maximum stresses change slightly at the ends and middle of the blades to 742 MPa and 415 MPa respectively, but this occurs in a small region of the device. This means there is no significant deviation in the maximum stress in the tool between the deployment step and the cutting step for the cutting forces investigated here. The retraction was not investigated numerically here because, absent an appropriate superelastic VUMAT model, it was not possible to accurately predict the recovery. The predicted recoverability of the device was therefore based on the peak stress and the observations of the model device.

### E. Experimental Results

**Fig. 9.**
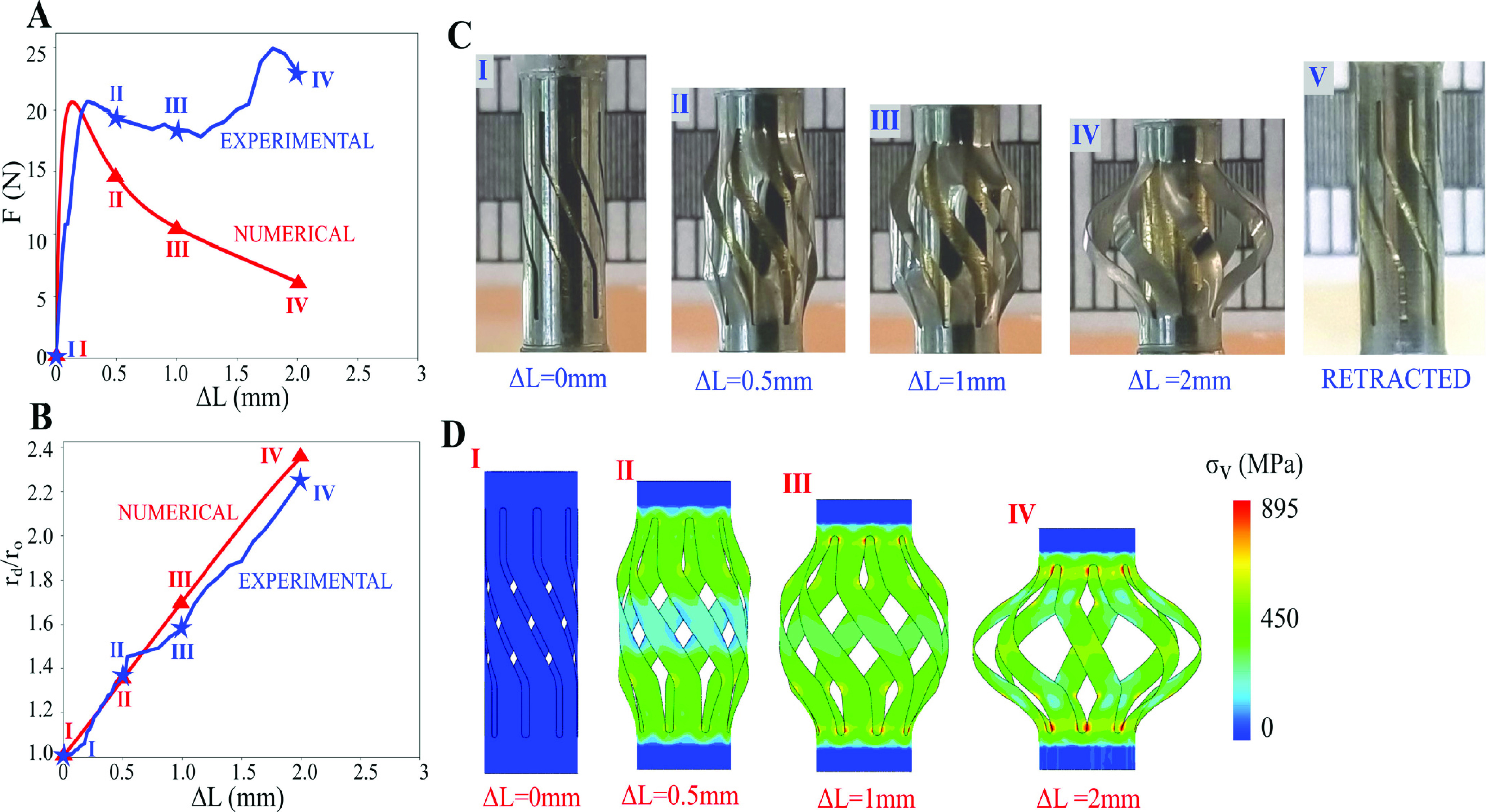
A) Force vs displacement and B) expansion ratio vs displacement of the NiTi device during deployment. C) Shows the progressive deployment of the device achieved experimentally and D) shows the progressive deployment of the device achieved numerically. Snapshot points are indicated in the plots.

The deployment reaction force and expansion ratio of the device are shown in Fig. 9 along with snapshots from the deployment. The device reaches a peak force of $F=21N$ at a displacement of $\Delta L=0.4 mm$, and achieves an expansion ratio of 2.3 at a compression of $\Delta L=2 mm$. It is predicted numerically for a device with a $75~\mu \text{m}$ wall thickness that there will be a peak force of $F=20.6N$ at a displacement of $\Delta L=0.3 mm$ and an expansion ratio of 2.4 at a compression of $\Delta L=2 mm$. This means that the experimental peak force is within 2% of the numerical prediction, providing a strong validation of the numerical response. The discrepancy between the numerical and experimental results at large displacement can be attributed to differences in the mechanical properties of the constituent NiTi, which are highly dependent on the exact composition. An isolated test on the tube material should be performed to validate the constituent properties, although this is outside the scope of this study. Additionally, it is likely that the shell elements used here are unable to capture geometric hardening due to local thickening of the shell, although it is still preferable to use shell elements to avoid complications with 3D elements for high aspect ratio parts. It should also be noted that the numerical prediction shown in Fig. 9 is different from the ones indicated above, which are all shown for a $50~\mu \text{m}$ wall thickness device.

## V. Discussions

### A. Role of Instabilities

The new biopsy device operates using coradial blades that are deployed via buckling. By changing the geometry and actuation of the tool, we are exploring the buckling deformation space of this design. The closest analytical approximation of this behavior is in the combined compression and torsion buckling of a helical spring [17], but this design replaces the cylindrical spring with a flat blade. The resulting deflection of the blades is a combined lateral/torsional buckling of the pre-bent blade [18]. This deformed state largely depends on the ratio between the helical displacement angle $\phi $ and the blade angle $\beta $. For the low twist state where $\phi < 90^{\circ }-\beta $, the blades will have an axial load and a bending moment, which will cause them to laterally buckle and twist, as is observed for small helical displacement angles (Fig. 6b). For the high twist state where $\phi >90^{\circ }-\beta $, the angled blades will similarly experience an axial and bending moment but in the opposite sense, which will cause them to buckle laterally but twist in the opposite direction. For the intermediate state where $\phi \approx 90^{\circ }-\beta $, the blades will purely laterally buckle and will undergo no twisting, as is observed for some loading cases (Fig. 6b, c). These buckled and twisted blade shapes also determine the expansion ratio of the tool, with designs that more purely laterally buckle achieving larger expansion ratios. Although these buckled shapes can in principle be predicted analytically, developing an analytical model is outside the scope of this work. It should be noted that these buckling behaviors do not directly correlate with the cutting ability of the tool.

The reaction force of the tool also directly depends on the buckling behavior of the blades but correlates with the blade angle and blade length. It is observed in Fig. 5 that as the length of the tool is increased, the reaction force drops in magnitude. This can be explained in the context of Euler buckling theory, which states that the maximum force required to buckle a column is inversely proportional to the square of the length of the column ($F\propto L^{-2}$). Shorter tool lengths ($L\le 10 mm$) also generally result in a stable, symmetric buckled state, although this is not explicitly guaranteed for all actuation angles $\phi $. For longer length beams, the buckled state is no longer symmetric, and the buckled shape of the blades is not easily predictable (Fig. 4a). It is also observed in Fig. 5c that there is a strong increase in the peak reaction force as the blade angle is increased. This is because as the blades become more aligned with the direction of applied load, they have a stiffer loading response, and a correspondingly larger drop in the peak force.

It is important to note that the peak deployment force in Fig. 5 represents a maximum force case for an ideal tool. If there is any pre-bend introduced in the blades, intentionally or otherwise, the peak force will be significantly reduced [19], as is traditionally observed in beam buckling. For significantly bent blades, there will be no peak force followed by a load drop at all. Instead, the deployment force will plateau at the force observed for large displacements, $\sim 3N$ for the case of $L=10 mm$ and $\beta =60^{\circ }$. This ~1/3 reduction in peak force can be used as a design consideration, especially if thicker blades need to be used to improve the cutting resistance.

### B. Cutting Ability

The effectiveness of the biopsy tool design depends on its ability both to resist large deflections during cutting and to maintain the blade orientation coradial to the circumference of the duct. The cutting stiffness has a strong dependence on each of the design parameters studied here. The stiffness as a function of $L$ in Fig. 7a suggests that the intermediate tool ($L=10 mm$) is the most effective because it has the lowest relative deflection, although this is complicated by the fact that each design was taken to a $\Delta L=2 mm$ compression. Further, the non-uniform buckled state of the longer designs makes them ineffective due to their unreliable blade deployment. The cutting stiffness as a function of $\phi $ in Fig. 7b suggests that increasing the helical deployment angle would lead to a more effective cutting device, but the poor alignment of the blades for large $\phi $ (i.e., parallel to the tool axis) suggests that it would lead to an ineffective cutting device. The stochastic changes in cutting stiffness as a function of $\beta $ in Fig. 7c arise due to the switching between different buckled states. It is difficult to definitively say what the most effective cutting angle would be, but it generally falls somewhere between $45^{\circ }< \beta < 75^{\circ }$, as the small $\beta $ leads to a localized buckling and the large $\beta $ does not lead to a well oriented blade (i.e., coradial with the duct circumference). There is not a considerable difference in the blade angle in the deployed state between the $L=8 mm$ and the $L=10 mm$ designs.

In the results obtained for the deflection of the blades, the maximum force applied on the blades has been taken to be $1N$ [20]. In the modelling of this tool, it is concluded that the threshold force above which the blades start to undergo large deflection is $0.2N$ for a force applied along the axis of the tool that purely acts on the edges of the blades. Determining a reasonable maximum force during cutting for the tool is difficult because there is no experimental data available for the mechanical properties of human biliary tissue [21]. The human coronary artery is generally similar in size and toughness to the bile duct and may provide some estimate of mechanical properties. The failure stresses and strains for healthy human coronary arteries were $\sigma _{ult}=1.44\pm 0.87 MPa$ and $\varepsilon _{ult}=0.54\pm 0.25$ respectively [22], but experimental data on the deformation characteristics of human coronary arteries are very limited [22]. Further experimentation of the actual tool is needed to verify its cutting ability.

### C. Optimal Tool Design Based on Numerical Modeling

From the results of the numerical analysis, a design with an acceptable combination of parameters was selected wherein the tool length is $L=10 mm$ and the blade angle is $\beta =60^{\circ }$. This ensures that the peak deployment force, which was $\sim 8.6N$ for this design, does not exceed the maximum force to cause bending or kinking in the support tube, the threshold for which is $30N$. This exact deployment force will change depending on the radius and overall wall thickness of the tool, which again are $3.5 mm$ and $50~\mu m$ here respectively. This means that it may be necessary to use different design parameters than those selected here depending on the availability of tube material. The helical deployment angle $\phi $ can vary without affecting the peak actuation force, but it should be taken to be $\phi \approx 34^{\circ }$ to ensure that the blades remain properly oriented to cut the tissue. All the designs studied here ensure a sufficient minimum expansion of the tool of at least 1.8x, but the design chosen has an expansion ratio over 3.0 at an applied compression of $\Delta L=2 mm$.

### D. Translation to Clinical Use

Our chosen pathway to clinical testing consists of 1) improving the performance of the tubular biopsy device, 2) testing the device ex vivo, 3) adding sub-assemblies to the tool, 4) testing in a realistic setting such as a phantom and 5) validating the numerical model performance in a realistic environment. We are confident that the device outer diameter of 3.5 mm and length of 10 mm can be used in adults for the ERCP procedure using clinically available instruments. Our experience during clinical trials using a custom ultrathin scanning fiber endoscope and small biopsy forceps side-by-side within a flexible tube of 3.6 mm outer diameter, which constitutes an internal rigid tip length of 10 mm, can be manipulated out of the endoscope and into the small intestine of 25 mm diameter to enter the pancreatobiliary opening during ERCP [23], [24]. Prior to passing the 3.6 mm tube through the main bile duct, the indeterminate biliary stricture is located using fluoroscopy and at site of the constriction a balloon catheter is used to temporarily expand the collapsed lumen. The biocompatibility of NiTi superalloys have been established in predicate devices and the required testing procedures are less stringent when tissue contact is brief, such as during a biopsy procedure [25].

Performance improvements to the device will require refining the fabrication to increase the sharpness of the blades. This can be done by offsetting and angling the focused laser beam during cutting. The sharpened device will be tested by actuating the device to protrude the blades then cutting tissues ex vivo to determine both the forces required for cutting and the cutting resistance of the blades. This would mirror the cutting step of the model (Fig. 7). Serration can be added to the blade edges to allow for piercing and tearing of the tissue, which may be necessary to improve the cutting ability with the simple axial motion of the device. Once the cutting forces required of the device can be determined, it will be possible to create more realistic numerical models.

Once tissue fragments can be generated, the device can be incorporated into a sub-assembly that will produce a pre-determined twist of the biopsy device when being compressed with guidewire. The assembly is expected to operate using a cam and plunger system that will follow a non-linear path to impart a twisting motion of the distal end of the biopsy device relative to the stationary proximal end of the device. This is shown conceptually in Figure 1. The trajectory of the twist will be chosen that minimizes the required tension on the guidewire and maximizes the recoverability (Fig. 5). The sub-assembly that holds the proximal end of the device will be created from a slightly modified pusher tube that is routinely used in interventional procedures. This functional biopsy device assembly will be tested in a phantom of the biliary system, which will be made of a silicone rubber that possesses tissue-like mechanical and optical properties. The synthetic tissue will be dyed so that the location of the cut fragments can be tracked [26]. Here, the capture system for the tissue fragments can additionally be tested, which will be facilitated either using additional fluid suction through the pusher tube by a syringe or through the addition of a thin sheath over the biopsy device.

The final design challenge is the hand piece, i.e. the manual deployment piece for the operator, which will have mechanisms to grip the guidewire (e.g. a pin vise) and then produce a measured amount of axial displacement to compress the biopsy device and expose the blades (e.g. via a ratcheting mechanism). Manual deployment of the device will be tested in the phantom and ex vivo tissues to verify that the complete system can produce tissue biopsies from small ducts. Human testing will require radiopaque contrast enhancers on the biopsy device to ensure sufficient contrast during x-ray fluoroscopy; this is the standard imaging procedure for interventional procedures using guidewires.

Clinical operation of the device will consist of the following steps. 1) The guidewire and radiopaque are inserted to navigate the biliary tree and find the stricture. 2) The stricture is expanded, if necessary, with a balloon catheter. 3) The custom guidewire with a ball end is inserted and aligned with the stricture of interest. 4) The tubular biopsy device is threaded over the custom guidewire. 5) The hand piece is used to pull the guidewire to the predetermined setting to deploy the blades. 6) The entire system is sharply pulled to cut the tissue. 7) The tension in the biopsy device is released. 8) The tissue fragments are collected. 9) The entire device is removed. 10) The tissue is sent to pathology for diagnosis.

Once the system has been tested in a realistic setting (either ex vivo or in vivo), the numerical model can be revisited to determine its efficacy in predicting the device behavior. Given the stiffness of the device constituent material (NiTi) and the compliance of the tissue, it is unlikely that there will be an effect on the device expansion at small displacements. This has been validated by preliminary tests in a tissue phantom. However, the confinement effect created by the surrounding tissue may change the buckling mode of the device at large deflections or during cutting, so to ensure there will be no spurious behavior, the device will need to be modeled along with the tissue. This will inevitably lead to many numerical challenges, and as such is beyond the scope of this study but is nevertheless important to investigate. It is unlikely that the fluid environment of the device will have a significant effect on its expandability, but it will likely affect the cutting performance due to various complications of friction and tissue hydration. This will be part of future investigations into the general cutting ability of the device.

## VI. Conclusion

We have outlined a design and corresponding numerical model for a novel biopsy tool that can be used in collecting tissue samples from the bile duct during an ERCP procedure. This model was carried out for the actual size device and implemented using the material properties for NiTi. Through the numerical analysis, we have clearly demonstrated the ability of the tool to expand and reach the inner lining of the bile duct. We have also clearly shown that the force required to deploy the tool is realistic and can be achieved manually with standard ERCP guidewires and catheters.

We have demonstrated the accuracy of the deployment and expansion of the device experimentally using a to-scale NiTi device fabricated using laser micromachining. Further work is required to test the cutting ability of the device in an ex-vivo or synthetic bile duct phantom. The sharpness and shearing ability of the tool blades are unknown at this point, and careful studies may be needed to fully quantify the cutting forces required to section tissue from the bile duct lining.

To obtain more realistic numerical results, the tool should be modeled as the part of a whole system. This can include the end effectors and grippers that will deploy the tool and a coaxial cable sleeve passing through the tool, but most importantly it should include cutting into the biliary luminal wall surrounding the tool. This numerical model would be highly non-trivial and is an area for further exploration. The quantity of tissue sample that can be obtained using this design can only be determined by creating the design in a bile duct or equivalent surgical phantom [23]. Therefore, further work is required both experimentally and numerically to quantify the cutting ability before the new design studied can be used in place of the conventional tools.
